# Inhibitory Effect on ***β***-Hexosaminidase Release from RBL-2H3 Cells of Extracts and Some Pure Constituents of Benchalokawichian, a Thai Herbal Remedy, Used for Allergic Disorders

**DOI:** 10.1155/2014/828760

**Published:** 2014-12-16

**Authors:** Thana Juckmeta, Pakakrong Thongdeeying, Arunporn Itharat

**Affiliations:** ^1^Faculty of Medicine, Thammasat University, Rangsit Campus, Khlong Luang, Pathum Thani 12120, Thailand; ^2^Department of Applied Thai Traditional Medicine, Faculty of Medicine, Thammasat University, Rangsit Campus, Khlong Luang, Pathum Thani 12120, Thailand; ^3^Center of Excellence on Applied Thai Traditional Medicine Research (CEATMR), Faculty of Medicine, Thammasat University, Rangsit Campus, Khlong Luang, Pathum Thani 12120, Thailand

## Abstract

*Introduction.* Benchalokawichian (BCW), a Thai traditional herbal formulation, has long been used as antipyretic and to treat skin disorders. It comprises roots from five herbs: *Ficus racemosa*, *Capparis micracantha*, *Clerodendrum petasites*, *Harrisonia perforata*, and *Tiliacora triandra*. This polyherbal remedy has recently been included in the Thailand National List of Essential Medicines (Herbal Products list). *Methodology.* A Bioassay-guided fractionation technique was used to evaluate antiallergy activities of crude extracts, and those obtained by the multistep column chromatography isolation of pure compounds. Inhibitory effect on the release of *β*-hexosaminidase from RBL-2H3 cells was used to determine antiallergic activity. *Results.* Two pure compounds from BCW formulation showed higher antiallergic activity than crude or semipure extracts. Pectolinarigenin showed the highest antiallergic activity, followed by *O*-methylalloptaeroxylin, with IC_50_ values of 6.3 *μ*g/mL and 14.16 *μ*g/mL, respectively. Moreover, the highest activities of pure compounds were significantly higher than chlorpheniramine (16.2 *μ*g/mL). *Conclusions.* This study provides some support for the use of BCW in reducing itching and treatment of other skin allergic disorders. The two isolated constituents exhibited high antiallergic activity and it is necessary to determine their mechanism of action. Further phytochemical and safety studies of pure compounds are required before development of these as antiallergy commercial remedies.

## 1. Introduction

Allergic diseases are manifested as hyperresponsiveness to allergenic environmental substances in the various target organs of the body (skin, nose, lung, gastrointestinal tract, etc.) and involve both IgE-mediated and non-IgE-mediated components [1, 2]. Exposure to allergenic materials results in production, by B cells, of a multitude of antibodies, collectively called immunoglobulins (Ig) that are antigen-specific. The allergic reaction starts when immunoglobulin E binds to specific receptors (FcRI) on the surface of mast cells and basophils [3, 4], which in turn induces degranulation of the cells and release of mediators such as histamine, leukotrienes, serotonin, and platelet activating factors [5–7]. Histamine is the main cause of many of the symptoms of allergies, such as runny nose, sneezing, and itching. Histamine also contributes to the progression of allergic-inflammatory responses by enhancement of the secretion of proinflammatory cytokines [8]. Although antihistamines are the first drugs of choice for treatment of many types of allergic disorders, they do have certain side effects. A large proportion (70–95%) of the world's population still relies on herbal medicines for primary health care [9]. Therefore, there is a continuous search for newer and better drugs for allergy treatment, including evaluation of traditional herbal remedies [10]. Since IgEs play an important role in the allergic reaction, it has been suggested that the way to treat and prevent allergic diseases is to block the activity of IgE response [11–13].

Benchalokawichian (BCW) is a Thai traditional medicine formulation containing parts from roots of five plants in equal amounts:* Ficus racemosa* Linn. (Moraceae),* Capparis micracantha* DC. (Capparidaceae),* Clerodendrum petasites* S. Moore. (Lamiaceae),* Harrisonia perforata* Merr. (Simaroubaceae), and* Tiliacora triandra* Diels. (Menispermaceae). It has long been used for relief of fever and to treat skin rash. This formulation is included in the Thailand National List of Essential Medicines [14]. It has also been used for prevention of influenza H1N1 infections and in recent years this remedy has been used to treat acne, skin rashes, and other similar skin disorders.

The polyherbal formulation BCW has not been systematically studied before, but there are numerous previous reports on the chemical constituents of some individual plants in this herbal remedy.* Harrisonia perforata* leaves, fruits, braches, and roots were shown to contain several chromones, limonoids, triterpenoids, and prenylated polyketides including harrisotone A–E, haperforine A, haperforine E, 12-desacetylhaperforine A, haperforine C2, haperforine F, haperforine G, Foritin, harrisonol A, peucenin-7-methylether,* O-*methylalloptaeroxylin, perforatic acid, eugenin, saikochromone A, greveichromenol, and perforamone A–D [15]. Other reported constituents are *β*-sitosterol, obacunone, herteropeucenin-7-methyl ether, perforatic acid and harrisonin [16–19], harperforatin, harperfolide, and harperamone [20].* Tiliacora triandra* has been reported to contain alkaloids, especially bisbenzylisoquinoline alkaloids, including tiliacorinine, tiliacorine, nortiliacorinine, and others [21–23].* Ficus racemosa* has been reported to contain tannins, flavonoids, coumarins, phenolic compounds, glycosides, and phytosterols [24, 25].

Currently the antipyretic and anti-inflammatory activities of BCW have only been studied* in vivo* in rats [26, 27]. The antimicrobial activity of ethanolic and water extracts of BCW has recently been reported [28, 29]. There are no other* in vitro* studies on antioxidant, antiallergy, or anti-inflammatory activities on BCW. However, a recent study on fruits of* H. perforata* has demonstrated that organic extracts exhibited high antioxidant activity by the DPPH method but failed to show any cytotoxicity against human myelogenous leukemia (K562) and human cancer (SGC-7901), cell lines* in vitro* by the MTT method [30]. Another report has also described the antioxidant activity in extracts of fruits of* H. perforata* by the DPPH method [31]. The results of* in vivo* rats suggest that* H. perforata* bark aqueous extracts does not cause acute and subchronic toxicities [32].

Benchalokawichian remedy has yet not been thoroughly studied, either* in vitro* or* in vivo*. Its recent inclusion in the Thailand National List of Essential Medicines has encouraged us to carry out systematic phytochemical and bioactivity assessment of crude extracts. Singharachai et al. studied morphological characters including macroscopic, microscopic examination, and pharmacognostic parameters and investigated 3D-HPLC fingerprint profile [33]. We also report the use of bioactivity-guided isolation of semipure and some pure constituents from this polyherbal remedy. In this preliminary study we have concentrated on antiallergic activity and on limited number of pure compounds. It is hoped that furthermore detailed studies of this type, along with safety studies in animals, will provide data that may allow the investigation of its clinical efficacy in controlled clinical trials for some of the conditions it is currently used for in Thailand by Thai Traditional medicine (TTM) practitioners.

## 2. Materials and Methods

### 2.1. Chemicals, Reagents, and Instrumentation

RBL-2H3 Rat basophilic leukemia cell line was from American Type Culture Collection (ATCC CRL-2256, VA, USA); fetal bovine serum (FBS), trypsin-EDTA, and trypan blue were purchased from Gibco (OK, USA). Minimum essential medium (MEM), penicillin-streptomycin (P/S), and phosphate buffer saline (PBS) were purchased from Biochrom (MA, Germany). 4-Nitrophenyl* N-*acetyl-*β*-D-glucosaminide (PNAG), antidinitrophenylated bovine albumin (DNP-BSA), anti-DNP IgE (monoclonal anti-DNP), ketotifen fumarate, and chlorpheniramine were purchased from Sigma (MO, USA). Dimethyl sulfoxide (DMSO) was purchased from Fluka (Munich, Germany). Calcium chloride dehydrate, magnesium chloride 6H_2_O, potassium chloride, and sodium carbonate were purchased from Merck (Darmstadt, Germany). Piperazine-*N,N*′-bis(2-ethanesulfonic acid) (PIPES) was purchased from Amresco (OH, USA). Sodium chloride and sodium hydroxide (analytical grade) were purchased from Univar (Ajax Finechem, Australia). Sodium bicarbonate was purchased from BDH (Poole, U.K.). The solvents for analysis, hexane, chloroform, ethyl acetate, and methanol (analytical grades) were purchased from RCI Labscan (Bangkok, Thailand). Sterile water was obtained by purification using a Milli Q system from Millipore (Bedford, MA, USA). Silica gel 60 grade numbers 1.07734 and 1.09385 (70–230 mesh and 230–400 mesh) and TLC silica gel 60 F_254_ were purchased from Merck (Darmstadt, Germany). Chromatographic column (4.5 × 54 cm, glass) was purchased from Becthai (Bangkok, Thailand). CO_2_ humidified incubator was purchased from Shellab (OR, USA). Laminar air flow cabinet was purchased from Boss tech (Bangkok, Thailand). Microplate reader was purchased from BioTek (VT, USA).

### 2.2. Plant Materials

Roots of five plants were collected from Dan-Chang, Suphan Buri Province, in Thailand in March 2012. Authentication of plant materials was by comparison against specimens deposited at the herbarium of Southern Center of Thai Medicinal Plants, Faculty of Pharmaceutical Science, Prince of Songkla University, Songkla, Thailand.

### 2.3. Preparation of the Extracts

The roots from each of these five plants were cleaned, cut in small pieces, and dried at 50°C 24 h. Each dried plant material was powdered using an electric grinder (40 mesh particle size). Five plant powders in the same ratio were mixed to provide the BCW remedy. BCW and each of the five plants were macerated with 95% ethanol, filtered with whatman number 1 and solvent removed using a rotary evaporator under reduced pressure (40°C) to obtain the dry ethanolic extracts. These extracts were further dried to constant weight in a vacuum desiccator. All extracts were kept at −20°C until required for further use.

### 2.4. Preparation of Semipure Extracts and Isolation of Pure Compounds

#### 2.4.1. Vacuum Liquid Chromatography (VLC)

Bioassay-guided fractionation was used to isolate the pure compounds by the following modified method of Tewtrakul and Itharat [34]. The BCW ethanolic extract (50.46 g) was subjected to silica vacuum liquid chromatography (VLC), using five solvent systems of increasing polarity; hexane (1500 mL), hexane : chloroform (1 : 1, 2000 mL), chloroform (2500 mL), chloroform : methanol (1 : 1, 2500 mL), and methanol (2000 mL). The VLC column was packed with silica gel 60 (mesh 230–400), the crude extract was applied on top of the column, then they were eluded with these five different solvents. The solvent in each fraction (fractions 1–5) was removed by rotary evaporator (40°C), and the dry semipure extract further dried to constant weight.

#### 2.4.2. Further Purification by Gravity Feed CC to Isolate Pure Compound

The fraction 3 (chloroform, 5.02 g) which exhibited the highest antiallergy activity was rechromatographed by column chromatography (4.50 cm diameter and 54 cm length) using silica gel G (70–230 mesh), with gravity feed of the following solvents in sequence: hexane : chloroform (1 : 1, 500 mL); chloroform (300 mL); chloroform : methanol (9 : 1, 500 mL); chloroform : methanol (1 : 1, 200 mL); methanol (300 mL). Fractions (12 mL) were collected during elution with each solvent (the first and the last fractions, 500 mL per each fraction, were not collected). Each of total fractions collected was examined by TLC.

The fraction 12 (282.6 mg) was rechromatographed by column chromatograph (3.75 cm × 54 cm) of silica gel grade numbers 70–230 mesh, by eluting sequentially with solvents of increasing polarity; hexane : EtOAc (7 : 3, 1000 mL); hexane : EtOAc (1 : 1, 400 mL); hexane : EtOAc (3 : 7, 300 mL); EtOAc (700 mL); EtOAc : MeOH (1 : 1, 100 mL), and finally MeOH (200 mL). Fractions (5 mL) were collected for each eluting solvent and all fractions were examined by TLC (GF_254_).

#### 2.4.3. Identification of Compounds 1 and 2

TLC (silica gel 60 GF_254_ aluminium sheets, Merck) were used to demonstrate the purity of compounds, with three different solvent systems of varying polarity and detection with anisaldehyde reagent. The structures of the isolated compounds were determined by their NMR data [^1^H and ^13^C on a Varian Unity Inova 500 spectrometer (500 MHz for ^1^H; 125 MHz for ^13^C)], UV spectra [SPECORD S 100 (Analytikjena) spectrometer], and ESI mass spectra, both HRMS and LRMS, were obtained from a Agilent Technologies 1200 Binary LC System coupled to a Bruker microtof mass spectrometer.

### 2.5. Determination of Antiallergic Activity

Inhibitory effects on the release of *β*-hexosaminidase from Rat Basophilic Leukemia cell line (RBL-2H3) were evaluated by the following modified method [35]. RBL-2H3 cells were cultured in MEM medium supplemented with 15% fetal bovine serum (FBS), penicillin (100 units/mL), and streptomycin (100 *μ*g/mL). The cells were seeded in 24-wells plate (5 × 10^5^ cells/mL) and incubated to adhere at 37°C in 5% CO_2_ for 1.5 hour. RBL-2H3 cells were sensitized with anti-DNP IgE (antidinitrophenyl-immunoglobulin E) (0.45 *μ*g/mL), and incubated at 37°C in 5% CO_2_ for 24 h. The cells were washed with 400 *μ*L of Siraganian buffer (buffer A) [119 mM NaCl, 5 mM KCl, 5.6 mM glucose, 0.4 mM MgCl_2_, 1 mM CaCl_2_, 25 mM piperazine-*N,N*′-bis(2-ethanesulfonic acid) (PIPES), 0.1% bovine serum albumin (BSA), and 40 mM NaOH, pH 7.2]. An aliquot (160 *μ*L) of buffer A was added and incubation was continued for an additional 10 min at 37°C. The test sample (20 *μ*L) solution was added to each well and incubated for 10 min, followed by addition of 20 *μ*L of antigen (DNP-BSA, final concentration 10 *μ*g/mL) at 37°C for 20 min to stimulate cell degranulation. The supernatants were transferred into 96-well plate in 50 *μ*L/wells and incubated with 50 *μ*L of substrate PNAG (1 mM* p-*nitrophenyl*-N-*acetyl-b-D-glucosaminide) in 0.1 M citrate buffer (pH 4.5) at 37°C for 3 h. The reaction was stopped by adding 200 *μ*L of stop solution (0.1 M Na_2_CO_3_/NaHCO_3_, pH 10.0). The absorbance was measured with a microplate reader at 405 nm. The test samples were dissolved in dimethyl sulfoxide (DMSO), and Siraganian buffer was added for dilution (final DMSO concentration was 0.1%). The positive controls showed clear yellow color, whereas the negative control was colorless. The samples were pale yellow to colorless, representing the percentage of inhibition antiallergic activity. Chlorpheniramine was used by positive controls. The percentage of inhibition was calculated according to the following formula:

(1) $$\begin{array}{c} \%\;\;\text{Inhibition}=\left[1-\frac{\left(T-B-N\right)}{\left(C-N\right)}\right]\times 100. \end{array}$$

Control (*C*): DNP-BSA (+), Test sample (−); Test (*T*): DNP-BSA (+), Test sample (+); Blank (*B*): DNP-BSA (−), Test sample (+); Normal (*N*): DNP-BSA (−), Test sample (−).

### 2.6. Statistical Analysis

The results are based on three separate experiments. Each sample was analyzed in triplicate in any experiment. The activity values are expressed as mean ± SD. IC_50_ values were calculated using the Prism Program.

## 3. Results

### 3.1. Yield of Crude Extracts and Fractions (F1–F5)

Yields (% w/w) of 95% ethanolic crude extracts of BCW remedy and its constituent were low, in range of 2–4% (see in Table 1).

The ethanolic extract of BCW (50.46 g) was subjected to vacuum liquid chromatography (VLC) to obtained five semipure extracts (F1–F5). Fraction 4 (chloroform : methanol elution) showed the highest yield, followed by fraction 3 and fraction 5, with yields being 65.40%, 13.20%, and 10.52%, respectively. The yields are shown in Table 2. All extracts and fractions were test inhibitory effects on release of *β*-hexosaminidase. The fractions which showed highest antiallergy activity were further subjected to purification to isolate pure compounds.

### 3.2. Isolation and Identification of Isolated Pure Compounds 1 and 2

The semipure extract from fraction F3 of VLC was purified using silica gel chromatogram (70–230 mesh) using solvents of increasing polarity for elution, with gravity feed of solvents. This step of purification resulted in the isolation of two pure compounds. All collected fractions were examined by TLC using anisaldehyde (in H_2_SO_4_) as detection spray. Compound 1 appeared as a yellow spot on TLC plates on heating and compound 2 appeared as yellow spot with no heating required. Compounds 1 and 2 were further identified by ^1^H and ^13^C NMR, and the structure identity confirmed by mass spectroscopy; compound 1 was pectolinarigenin and compound 2 was* O-*methylalloptaeroxylin.

**Figure 1:**
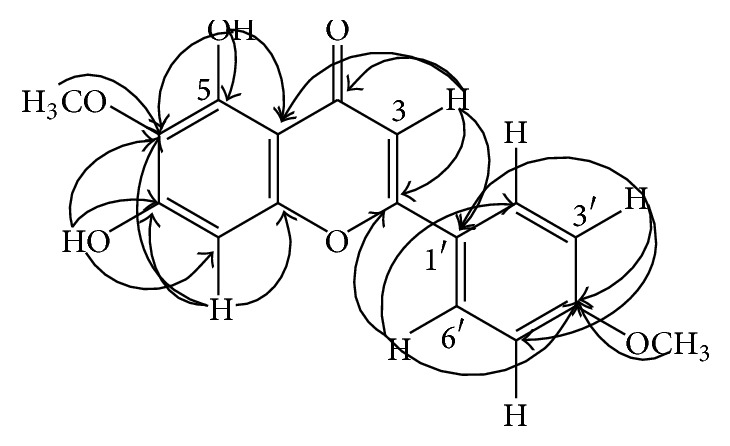
HMBC correlation of compound pectolinarigenin.

On recrystallization of material from methanol afforded a crystalline pale yellow solid (5 mg,) as compound 1 pectolinarigenin or 5,7-dihydroxy-6-methoxy-2-(4-methoxyphenyl)-4H-chromen-4-one (PubChem CID: 5320438). The molecular formula of compound 1 was proposed to be C_17_H_14_O_6_, as deduced from ESI mass spectra [m/z 315.0863; (M+H)+], ^1^H NMR (500 MHz, CDCl_3_), and the ^13^C NMR (125 MHz, CDCl_3_), 17 carbon signals observed, 10 of which correspond with 14 protons as observed from HMBC spectrum (Figure 1). NMR data is shown in Table 3.

**Figure 2:**
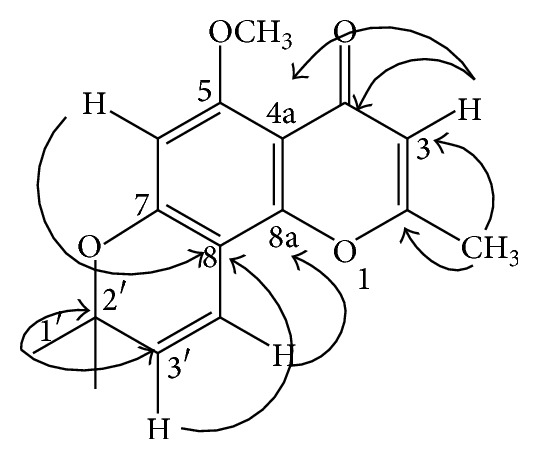
HMBC correlation of compound* O-*methylalloptaeroxylin.

The fraction 6 was a yellow solid (108 mg,) and was identified as compound 2* O-*methylalloptaeroxylin (PubChem CID: 441968). The molecular formula of compound 2 was proposed to be C_16_H_16_O_4_, as deduced from ESI mass spectra [m/z 273.1121; (M+H+)], ^1^H NMR (500 MHz, CDCl_3_), and the ^13^C NMR (125 MHz, CDCl_3_) in Table 4, 16 carbon signals observed, 7 of which correspond with 16 protons as observed from HMBC spectrum (Figure 2).

### 3.3. Inhibitory Effects on Release of *β*-Hexosaminidase

IC_50_ values of antiallergic activity against release of *β*-hexosaminidase in RBL-2H3 cell lines are shown in Table 5. The ethanolic crude extract from* H. perforata* exhibited the most potent antiallergic activity, followed by* F. racemosa* and the polyherbal BCW formulation (IC_50_ = 14.5, 27.7 and 39.8 *μ*g/mL, resp.).* C. petasites* showed only moderate activity (IC_50_ = 57.8 *μ*g/mL), whereas* C. micracantha* and* T. triandra* were inactive (IC_50_ > 100 *μ*g/mL).

The semipure extracts from fraction F3 and fraction F4 after VLC showed high inhibitory activity (IC_50_ values 17.9 and 19.6 *μ*g/mL, resp.). Other semipure extracts (F2 and F5) had moderate activity, whereas the hexane extract (F1) was not tested.

Pectolinarigenin and* O-*methylalloptaeroxylin exhibited the highest antiallergic activity, with IC_50_ values of 6.3 and 14.2 *μ*g/mL (20.1 and 51.8 *μ*M), respectively. These two compounds show higher inhibitory effect than chlorpheniramine, positive control (IC_50_ = 16.2 *μ*g/mL, 58.8 *μ*M).

## 4. Discussion and Conclusions

This study has isolated for the first time two pure compounds from the ethanolic extract of BCW formulation and identified them as pectolinarigenin (compound 1) and* O-*methylalloptaeroxylin (compound 2), which have not presented on 3D-HPLC chromatogram in previous study [33]. Compound 2 has previously been isolated from branches of* H. perforata* [36, 37]. In a recent study Choodej et al. [20] have investigated the effects of compound 2 on NO production in LPS-stimulated macrophages and showed it had potent activity, with IC_50_ value of 66.41 ± 5.21 *μ*M. Although this structure was described more than ten years ago, research on inhibitory effect against release of *β*-hexosaminidase in RBL-2H3 cell lines has not been described to date. This is, therefore, the first report on antiallergic effect of compound 2.

Pectolinarigenin (compound 1) had never been reported in any of the constituent plants of BCW formulation. However, this compound has been isolated from several other plant families; Scrophulariaceae [36–38], Compositae [39–42], Bignoniaceae [43], Fabaceae [44], Verbenaceae or Lamiaceae [45–49]. Interestingly, pectolinarigenin has been isolated from* Clerodendrum spp.* [46, 47, 49], related to* C. petasites*, a constituent plant of the BCW formulation. In another study, the ethyl acetate fraction of* Cirsium chanroenicum* (Compositae) also showed strong inhibition of COX-2-mediated PGE2 and 5-LOX-mediated LT production* in vitro*. It showed the same inhibitory effect in several animal models of inflammation/allergy, such as arachidonic acid-induced mouse ear edema, carrageenan-induced mouse paw edema, and passive cutaneous anaphylaxis [39]. Pectolinarigenin has also been shown to have hepatoprotective activity in a rat model of hepatic injury caused by D-galactosamine (GalN) mainly* via* SOD antioxidant mechanism [40]. Inflammation mediators and reactive free radicals do have a role in the pathophysiology of allergic disorders, and, therefore, anti-inflammatory and antioxidant compounds often exhibit antiallergic activities.

**Figure 3:**
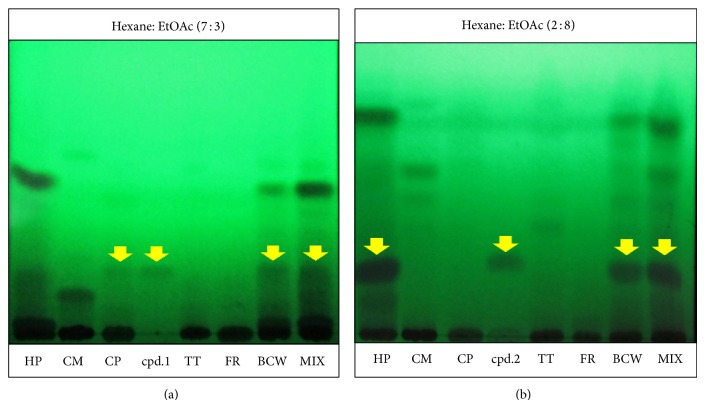
TLC chromatogram of five plants, BCW extract, mix of all extracts and pure compounds: compound 1 (a) and compound 2 (b). Note: CM =* Capparis micracantha* DC., CP =* Clerodendrum petasites* S. Moore, FR =* Ficus racemosa* Linn., HP =* Harrisonia perforata* Merr., and TT =* Tiliacora triandra* Diels.

**Figure 4:**
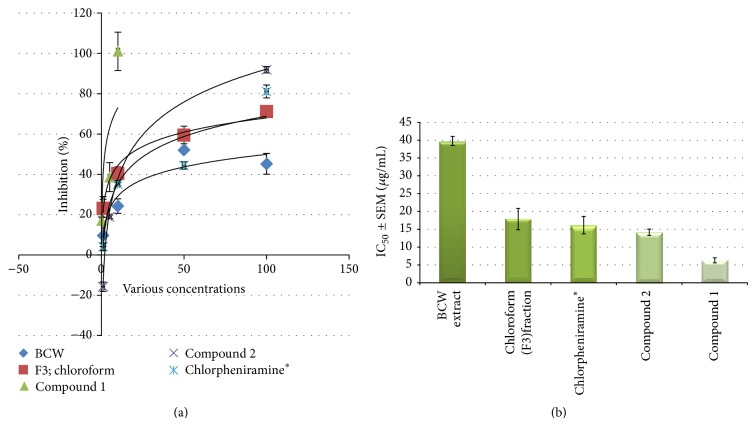
Effect of antiallergic activity against release of *β*-hexosaminidase in RBL-2H3 cell lines comparison of the BLW crude extract, fraction 3, pure compounds, and positive controls. (a) % is inhibition at various concentration. (b) Data are present as the IC_50_ (Mean ± SEM, *μ*g/mL) and ∗ = positive control.

The study on TLC chromatogram of two compounds (Figure 3), we found that compound 1 (pectolinarigenin) is presented on* H. perforata*, whereas compound 2 (*O-*methylalloptaeroxylin) is presented on* C. petasites* in the same as previous reviews. Both of pure compounds show exhibited higher antiallergic activity than all fractions, BLW extract and positive control, represented in Figure 4. This preliminary study provides some supports for the use of BCW for treatment of allergic skin rash in Thai traditional medicine. This is the first report on the two isolated compounds from BCW ethanolic extract and their antiallergic activities. The ethanolic extract of BCW could be further developed into commercial formulations for the treatment of allergic dermal diseases, whereas the two pure compounds can serve as bioactive markers for the analysis and standardization of any new formulated products.

## Supplementary Material

The spectrum of compound 1 (pectolinarigenin) and 2 (O-methylalloptaeroxylin) which isolated from Benchalogawichien Extract (It composed with five Thai plants in equal proportion such as Ficus racemosa Linn., Capparis micracantha DC., Clerodendrum petasites S.Moore., Harrisonia perforata Merr., and Tiliacora triandra Diels).

## Figures and Tables

**Table 1:** Yields (%w/w) and voucher specimen number of Benchalokawichian formulation and constituent plants.

Sample	Family name	Voucher specimens	%Yield^*^
*Ficus racemosa* Linn.	Moraceae	SKP 117 06 18 01	4.18%
*Capparis micracantha* DC.	Capparidaceae	SKP 391 03 13 01	1.89%
*Tiliacora triandra* Diels.	Menispermaceae	SKP 114 20 20 01	3.92%
*Harrisonia perforata* Merr.	Simaroubaceae	SKP 178 08 16 01	2.78%
*Clerodendrum petasites* S. Moore	Lamiaceae	SKP 202 03 09 01	2.20%
Benchalokawichian remedy (Ha-Rak)		—	3.59%

^*^Extraction by maceration with 95% ethanol. Yields are %w/w of starting dry weight of powered roots or BCW formulation.

**Table 2:** Yields (%w/w) of fractions after VLC of the crude ethanolic extract of Benchalokawichian formulation.

Fraction; solvent system	%Yield^*^
1; hexane	0.06 (0.002)
2; hexane : chloroform	5.98 (0.214)
3; chloroform	13.26 (0.47)
4; chloroform : methanol	65.40 (2.35)
5; methanol	10.52 (0.38)

^*^Yield is %w/w of crude ethanolic extract (values in brackets are %w/w expressed of DPW).

**Table 3:** NMR spectrum data (500 MHz for ^1^H and 125 MHz for  ^13^C) of compound 1.

Position	^ 1^H (mult., *J* in Hz.)^a^	^ 13^C	HMBC (^1^H→^13^C)
2	—	164.2	
3	6.57 (s)	103.8	C-2, C-4, C-4a, C-1′
4	—	183.0	
4a	—	105.8	
5	—	152.1	
6	—	130.3	
7	—	154.9	
8	6.59 (s)	93.3	C-6, C-7, C-8a
8a	—	153.2	
1′	—	123.6	
2′	7.83 (dd, 8.9, 1.9)	128.1	C-2, C-4′, C-6′
3′	7.01 (dd, 8.9, 1.9)	114.5	C-1′, C-4′, C-5′
4′	—	162.6	
5′	7.01 (dd, 8.9, 1.9)	114.5	C-1′, C-3′, C-4′
6′	7.83 (dd, 8.9, 1.9)	128.1	C-2, C-2′, C-4′
5-OH	13.10 (s)		C-4a, C-5, C-6
7-OH	6.53 (s)		C-6, C-7, C-8
4′-OCH_3_	3.90 (3H, s)	55.5	C-4′
6-OCH_3_	4.04 (3H, s)	60.8	C-6

*Note.* Compound 1 in CDCl_3_; ^a^if not indicated; the integration of each proton signal equal to one proton.

**Table 4:** NMR spectrum data (500 MHz for ^1^H and 125 MHz for  ^13^C) of compound 2.

Position	^ 1^H (mult., *J* in Hz.)^a^	^ 13^C	HMBC (^1^H→^13^C)
2		162.5	
3	5.99 (s)	111.8	C-2, C-2, C-4, C-4a
4		177.5	
4a		108.5	
5		160.6	
6	6.29 (s)	96.4	C-4a, C-5, C-7, C-8
7		157.6	
8		102.3	
8a		154.3	
1′ × 2	1.47 (s)	28.2	C-1′, C-2′, C-3′
2′		77.9	
3′	5.55 (d, 10.0)	127.3	C-2′, C-8
4′	6.70 (d, 10.0)	115.3	C-7, C-8, C-8a, C-2′
2-CH_3_	2.28 (3H, s)	19.6	C-2, C-3
5-OCH_3_	3.91 (s)	56.4	C-5

*Note.* Compound 2 in CDCl_3_; ^a^if not indicated; the integration of each proton signal equal to one proton.

**Table 5:** Anti-allergic activity of the ethanolic extract, fractions and pure constituents from Benchalokawichian remedy.

Samples	%Inhibition at various concentrations						IC_50_ of Antiallergic activity
Crude, semipure extracts, and pure compounds	0.1	1	5	10	50	100	IC_50_ ± SEM, *μ*g/mL
							(*μ*M data for pure compound)
*Ficus racemosa* Linn.	—	15.0 ± 10.5	—	28.9 ± 9.1	63.6 ± 1.6	75.8 ± 4.3	27.1 ± 1.6
*Capparis micracantha* DC.	—	4.2 ± 2.7	—	9.5 ± 7.1	22.9 ± 8.9	32.3 ± 6.3	>100
*Tiliacora triandra* Diels.		−24.48 ± 5.7		−0.0 ± 1.8	11.7 ± 4.8	44.2 ± 4.6	>100
*Harrisonia perforata* Merr.	—	21.6 ± 5.3	—	42.4 ± 1.3	62.8 ± 0.0	78.5 ± 0.8	14.5 ± 0.1
*Clerodendrum petasites* S. Moore		−6.3 ± 5.1		7.9 ± 2.2	44.0 ± 1.4	85.0 ± 1.9	57.8 ± 1.4
Benchalokawichian (Ha-Rak)	—	9.7 ± 5.5	—	24.2 ± 3.6	52.1 ± 1.5	45.2 ± 5.1	39.8 ± 1.3

Fraction 1; hexane (F1)	—	—	—	—	—	—	NT
Fraction 2; hexane : chloroform (F2)	—	22.5 ± 0.4	—	30.4 ± 2.1	43.6 ± 0.8	53.0 ± 0.7	86.1 ± 1.1
Fraction 3; chloroform (F3)	—	23.1 ± 4.5	—	40.3 ± 3.4	59.5 ± 4.4	71.2 ± 1.7	17.9 ± 3.0
Fraction 4; chloroform : methanol (F4)	—	20.9 ± 7.7	—	38.9 ± 3.9	58.2 ± 2.7	70.5 ± 3.7	19.6 ± 3.4
Fraction 5; methanol (F5)	—	10.5 ± 7.6	—	26.0 ± 6.2	52.1 ± 1.5	63.9 ± 2.9	39.9 ± 1.9

Compound 1; pectolinarigenin	17.1 ± 1.7	25.1 ± 3.9	38.6 ± 7.2	101.1 ± 9.5	—	—	6.3 ± 0.7 (20.1 *μ*M)
Compound 2; *O-*methylalloptaeroxylin	—	−15.8 ± 2.3	18.9 ± 0.9	40.5 ± 0.0	—	91.9 ± 1.6	14.2 ± 0.9 (51.8 *μ*M)
Chlorpheniramine^a^	—	4.0 ± 1.9	—	35.1 ± 0.8	44.4 ± 1.8	81.1 ± 3.2	16.2 ± 2.5 (58.8 *μ*M)

^a^Positive control.

NT (—) means not tested.
