# Supplementary material for: Inhibitory Effect on β-Hexosaminidase Release from RBL-2H3 Cells of Extracts and Some Pure Constituents of Benchalokawichian, a Thai Herbal Remedy, Used for Allergic Disorders
Source: Evid Based Complement Alternat Med. 2014 Dec 16;2014:828760. doi: 10.1155/2014/828760 (PMC4280658; doi:10.1155/2014/828760)
Supplement: Supplementary file 1 — The spectrum of compound 1 (pectolinarigenin) and 2 (O-methylalloptaeroxylin) which isolated from Benchalogawichien Extract (It composed with five Thai plants in equal proportion such as Ficus racemosa Linn., Capparis micracantha DC., Clerodendrum petasites S.Moore., Harrisonia perforata Merr., and Tiliacora triandra Diels). [file 828760.f1.docx]

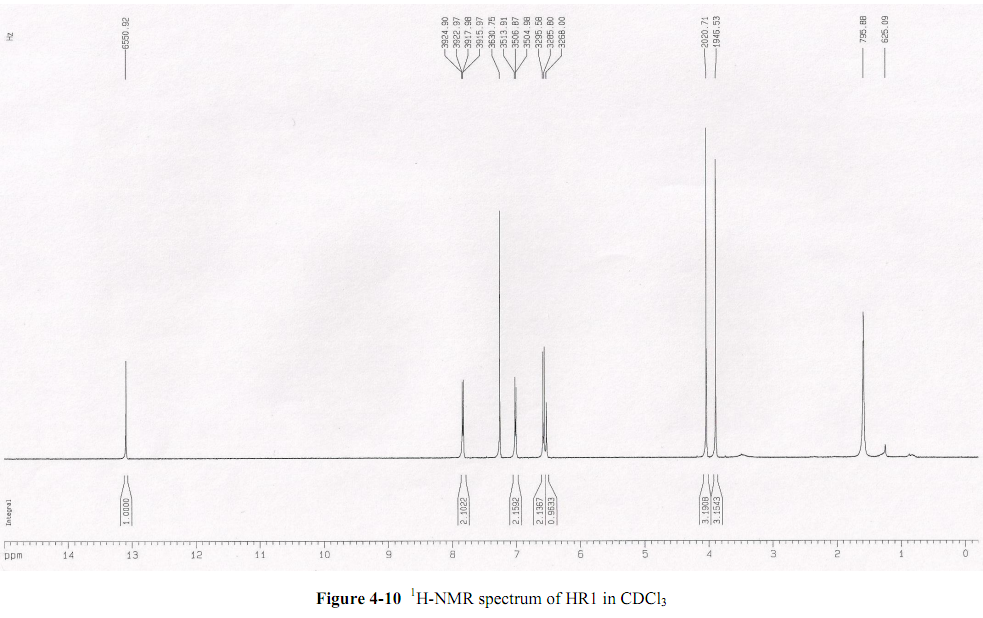


^1^H-NMR spectrum of compound1 in CDCl_3_


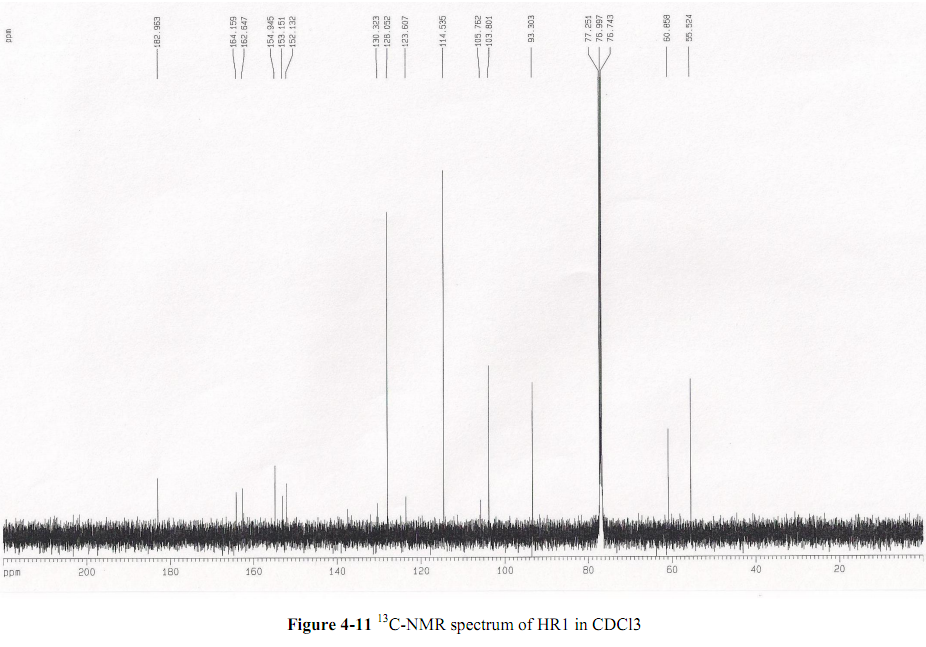


^13^C-NMR spectrum of compound1 in CDCl_3_


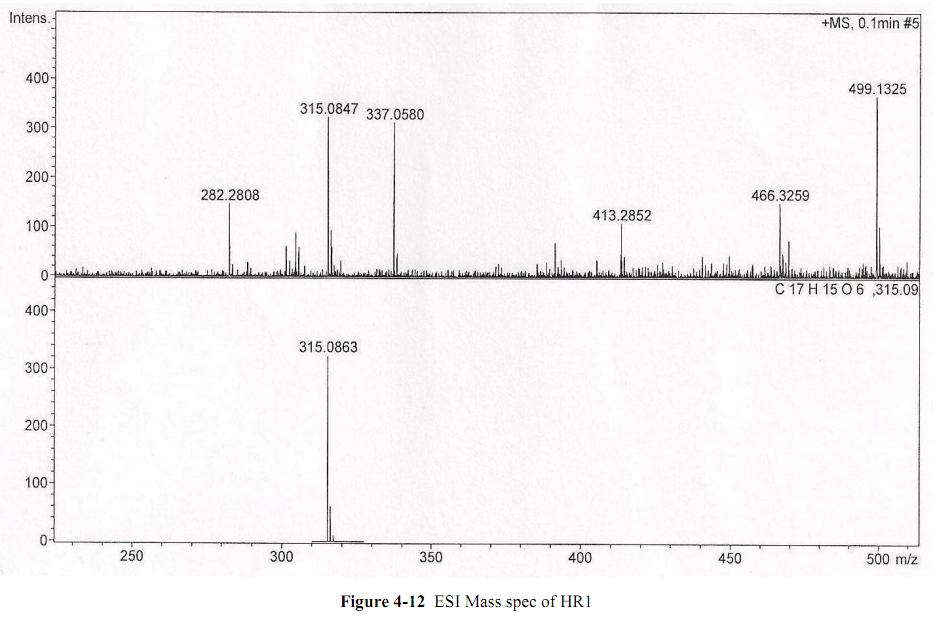


ESI Mass spec of compound1


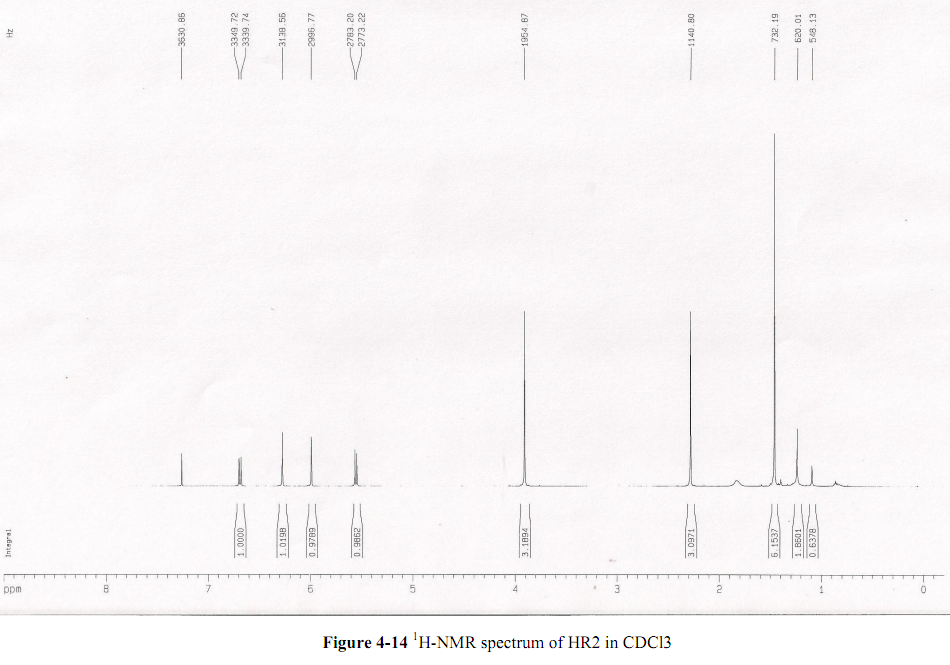
^1^H-^1^H-NMR spectrum of compound2 in CDCl_3_


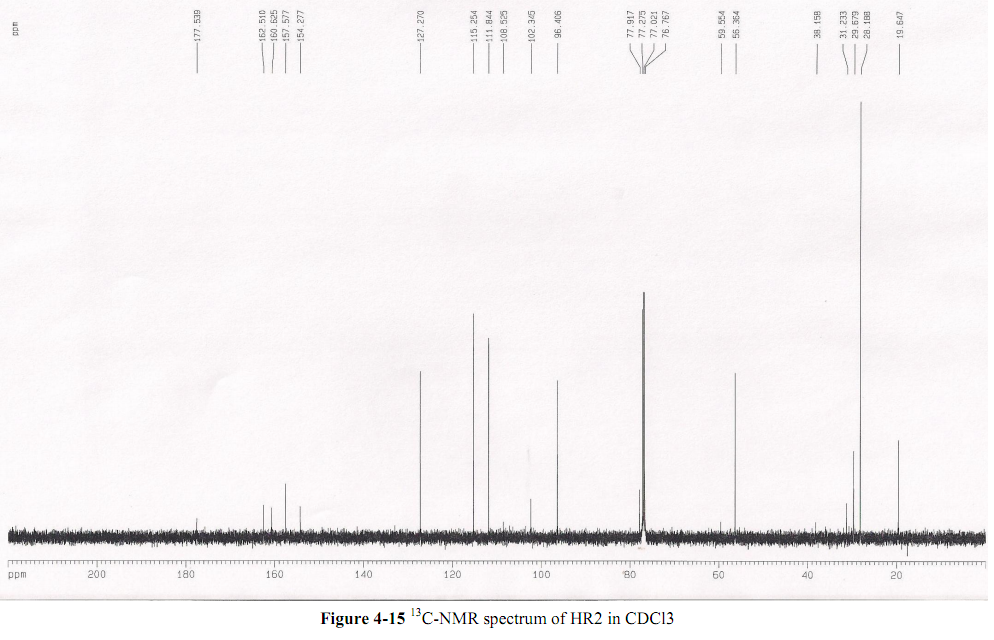
^13^C-NMR spectrum of compound2 in CDCl_3_


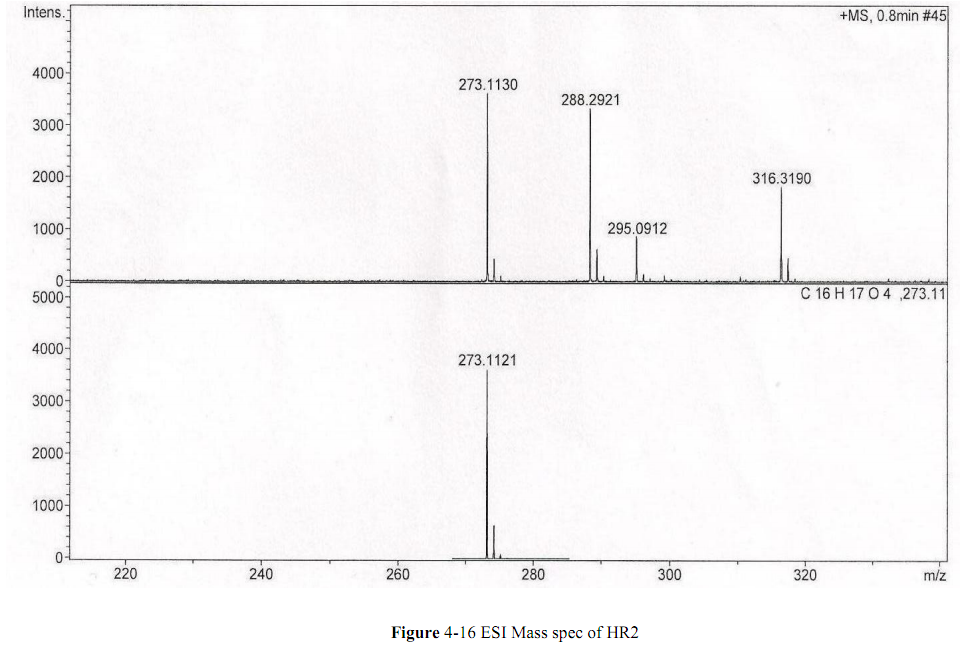


ESI Mass spec of compound2
